# Low levels of the AhR in chronic obstructive pulmonary disease (COPD)-derived lung cells increases COX-2 protein by altering mRNA stability

**DOI:** 10.1371/journal.pone.0180881

**Published:** 2017-07-27

**Authors:** Michela Zago, Jared A. Sheridan, Hussein Traboulsi, Emelia Hecht, Yelu Zhang, Necola Guerrina, Jason Matthews, Parameswaran Nair, David H. Eidelman, Qutayba Hamid, Carolyn J. Baglole

**Affiliations:** 1 Department of Medicine, McGill University, Montreal, Quebec, Canada; 2 Department of Pharmacology & Therapeutics, McGill University, Montreal, Quebec, Canada; 3 Research Institute of the McGill University Health Centre, McGill University, Montreal, Quebec, Canada; 4 Department of Pathology McGill University, Montreal, Quebec, Canada; 5 Department of Nutrition, University of Oslo, Oslo, Norway; 6 Department of Medicine, McMaster University, Hamilton, ON, Canada; University of Rochester Medical Center, UNITED STATES

## Abstract

Heightened inflammation, including expression of COX-2, is associated with chronic obstructive pulmonary disease (COPD) pathogenesis. The aryl hydrocarbon receptor (AhR) is a ligand-activated transcription factor that is reduced in COPD-derived lung fibroblasts. The AhR also suppresses COX-2 in response to cigarette smoke, the main risk factor for COPD, by destabilizing the *Cox-2* transcript by mechanisms that may involve the regulation of microRNA (miRNA). Whether reduced AhR expression is responsible for heightened COX-2 in COPD is not known. Here, we investigated the expression of COX-2 as well as the expression of miR-146a, a miRNA known to regulate COX-2 levels, in primary lung fibroblasts derived from non-smokers (Normal) and smokers (At Risk) with and without COPD. To confirm the involvement of the AhR, AhR knock-down via siRNA in Normal lung fibroblasts and MLE-12 cells was employed as were A549-AhR^ko^ cells. Basal expression of COX-2 protein was higher in COPD lung fibroblasts compared to Normal or Smoker fibroblasts but there was no difference in *Cox-2* mRNA. Knockdown of AhR in lung structural cells increased COX-2 protein by stabilizing the *Cox-2* transcript. There was less induction of miR-146a in COPD-derived lung fibroblasts but this was not due to the AhR. Instead, we found that RelB, an NF-κB protein, was required for transcriptional induction of both *Cox-2* and miR-146a. Therefore, we conclude that the AhR controls COX-2 protein via mRNA stability by a mechanism independent of miR-146a. Low levels of the AhR may therefore contribute to the heightened inflammation common in COPD patients.

## Introduction

Cigarette smoke is the leading risk factor for chronic obstructive pulmonary disease (COPD), an obstructive lung disease typified by the increased expression of inflammatory mediators such as interleukin-1 (IL-1) and cyclooxygenase-2 (COX-2) [1, 2]. COX-2 is an immediate/early gene that catalyzes the transformation of arachidonic acid (AA) into thromboxanes and prostaglandins (PG) such as PGE_2_. Inhibition of COX-2-derived PGE_2_ protects against the development of emphysema [3] which supports a role for chronic COX-2/PGs in the pathobiology of COPD.

Cigarette smoke contains more than 5000 different chemicals, many of which are capable of activating cellular signaling pathways such as the aryl hydrocarbon receptor (AhR). The AhR is a ligand-activated receptor/transcription factor that belongs to the basic helix-loop-helix Per-Arnt-Sim (bHLH/PAS) transcription factor family. The AhR is activated by polyhalogenated aromatic hydrocarbons (PAH) such as 2,3,7,8-tetrachlorodibenzop- dioxin (TCDD; dioxin) as well as polycyclic aromatic hydrocarbons (PAHs) such as benzo[*a*]pyrene (B[*a*]P)- a component of cigarette smoke [4, 5] After binding ligand, the AhR translocates from the cytoplasm to the nucleus, dissociates from cytosolic chaperone proteins and forms a heterodimer with the AhR nuclear translocator (ARNT). This AhR:ARNT complex then binds to a dioxin responsive element (DRE) to initiate the transcription of genes that comprise the AhR gene battery such as the phase I cytochrome P450 (CYP) enzyme CYP1A1.

Despite its notoriety as a mediator of toxicological responses, the AhR has now emerged as an important regulator of numerous physiological processes, including the suppression of inflammation caused by cigarette smoke [6, 7]. We have published that the AhR attenuates cigarette smoke-induced COX-2 expression *in vitro* and *in vivo* by controlling stability of the *Cox-2* transcript [4]. The AhR also controls COX-2 by mechanisms that involve RelB [6, 8], a component of the alternative NF-κB pathway. We have also shown that there is significantly less AhR protein expression in COPD lung fibroblasts [9], an important lung structural cell type and one of the main producers of PGs [10]. COPD-derived lung cells expresses higher COX-2 protein due to alterations in mRNA stability caused by poor induction of the microRNA (miRNA) miR-146a [11]. miRNA are single-stranded, non-coding, 22 nucleotide-long RNA that act post-transcriptionally to inhibit protein expression either by translational repression or enhanced mRNA degradation [12, 13]. *Cox-2* is a direct target of miR-146a, which has homology with the *Cox-2* 3’-untranslated region (UTR), the instability region of target mRNA that contributes to the rapid decay of the *Cox-2* transcript [11]. Whether low AhR levels contribute to heighted COX-2 expression in COPD due to regulation of miR-146a is not known.

To test this, we used primary lung fibroblasts from Normal, At Risk (Smoker) and COPD subjects, together with additional lung structural cells devoid of AhR expression, to show that the AhR controls COX-2 protein expression via alterations in *Cox-2* mRNA stability. However, the AhR did not control the induction of *Cox-2* mRNA or miR-146a. Instead, RelB- a component of the non-canonical NF-κB pathway- was required for transcriptional induction of both *Cox-2* mRNA and miR-146a in response to inflammatory stimuli. Our data continue to support that the AhR provides protection in the lung via pathways that are independent from its well-known toxicological roles associated with dioxin. Improved insight into the mechanistic relationship of pulmonary AhR may contribute to the development of novel, lung-targeted anti-inflammatory treatments for diseases such as COPD.

## Materials and methods

### Materials

All chemicals were purchased from Sigma (St. Louis, MO) unless otherwise indicated. Recombinant human and mouse IL-1β (rhIL-1β and rmIL-1β, respectively) were purchased from R&D Systems (Minneapolis, MN) at a concentration of 10 ng/ml. CH-223191 (1-methyl-N-[2-methyl-4-[2-(2-methylphenyl) diazenyl] phenyl-1H-pyrazole-5-carboxamide) was purchased from Tocris Bioscience (Minneapolis, MN). Actinomycin D (ActD) was purchased from Biomol (Plymouth Meeting, PA).

### Cell culture

The clinical features of the subjects from which the lung tissue was derived are as previously published [9, 14] but included lung tissue from individuals with COPD, subjects without COPD but who were current or former smokers (At Risk) or non-smokers without COPD (Normal). Derivation of primary human lung fibroblasts from these tissues is also as described [14, 15]. This study was approved by the Research Ethics Board of St Joseph’s Healthcare Hamilton and all patients gave written informed consent, Primary mouse lung fibroblasts were generated from AhR wild-type (*Ahr*^*+/+*^), AhR heterozygous (*Ahr*^*+/-*^) and AhR knock-out (*Ahr*^*-/-*^) mice as described [15]. All animal procedures were approved by the McGill University Animal Care Committee (Protocol Number: 5933) and were carried out in accordance with the Canadian Council on Animal Care. *Ahr*^*+/+*^ and *Ahr*^*+/-*^ fibroblasts show no significant difference in response to cigarette smoke or classic AhR ligands and are therefore used interchangeably as AhR-expressing cells [4, 6]. All fibroblasts (mouse, human) were used at the earliest possible passage and cultured under standard conditions. MLE-12 cells (ATCC, Manassas,VA) [16] were cultured in HITES medium (50:50, DMEM: Ham’s F-12) supplemented with 2% FBS, 2 mM L-glutamine, 10 mM HEPES, 1:100 insulin/transferrin/selenium supplement (Invitrogen) and antibiotics/antimycotics. Generation and characterization of A549-AhR knockout (A549-AhR^ko^) cells was accomplished by zinc finger nucleases (ZFNs) as previously described [17, 18].

### Preparation of cigarette smoke extract (CSE)

Research grade cigarettes (3R4F) with a filter were obtained from the Kentucky Tobacco Research Council (Lexington, KT) and CSE generated and standardized as previously described [4, 8, 18, 19].

### Analysis of gene expression

Total RNA was isolated using a Qiagen miRNeasy kit (Qiagen Inc., Valencia, CA) and purity measured using a Nanodrop 1000 spectrophotometer (Thermo Fisher Scientific, Wilmington, DE). Real time (qPCR) was performed with 1 μl cDNA and 0.5 μM primers as described [9]. Primer sequences for human *Cox-2* are TCACAGGCTTCCATTGACCAG (f) and CCGAGGCTTTTCTACCAGA (r). Sequences for human RelB are TGTGGTGAGGATCTGCTTCCAG (f) and GGCCCGCTTTCCTTGTTAATTC (r) and sequences for mousse RelB are CCTGTCTCCATATCCCTTCCTG (f) and CGCTGCAAAAGAGTCCAGTGA (r). Gene expression data were analyzed using the ΔΔCt method normalized to housekeeping (β-actin).

### Analysis of miR-146a expression

miRNA expression was assessed by two-step TaqMan® RT-PCR (Applied Biosystems, Carlsbad, CA) for miR-146a and U6 snRNA, a small nuclear RNA (snRNA) used as an internal control for miRNA analysis [20, 21]. miRNA expression was normalized to the U6 snRNA levels and fold-change was determined using 2^−ΔΔCt^ method as we have described [18, 22].

### Determination of Cox-2 mRNA stability

Cells were cultured until near confluence and switched to serum-free media for 24 hours. Then the cells were exposed to CSE for 3 hours followed by treatment with ActD (1 μg/ml), an inhibitor of RNA synthesis [23]. Total RNA was harvested and qPCR performed as described above to determine the remaining levels of *Cox-2* mRNA.

### Western blot

Total cellular protein was prepared using 1% IGEPAL lysis buffer [24] and 5–10 μg of protein were fractionated on SDS-PAGE gels and electro-blotted onto Immun-blot PVDF membrane (Bio-Rad Laboratories, Hercules, CA). Antibodies against AhR (1:5000; Enzo Life), RelB (1:1000; Santa Cruz) and COX-2 (1:1000) (Cayman Chemical, Ann Arbor, MI) were used to assess changes in relative expression.

### siRNA knock-down experiments

AhR knock-down was performed as recently described [19]. Briefly, cells were seeded at a density of 7.5 x 10^3^ cells/cm^2^ and transiently transfected with 60 nM siRNA against AhR (Santa Cruz, catalogue number sc-29655) or control siRNA (Santa Cruz, catalogue number sc 37007) according to the manufacturer’s instructions. For RelB knock-down, normal fibroblasts (non-smoker) were seeded at 1–2 x 10^4^ cells/cm^2^ and transfected with 40 nM of siRNA against RelB (Santa Cruz, Catalogue number sc-36402) or non-targeting control siRNA (Santa Cruz, Catalogue number sc-37007) as described [14]. Six hours after the transfection, the cells were switched to serum-free MEM. On the next day, cells were treated with IL-1β and RNA or protein collected for further analysis as described above. Verification of target knockdown was done by Western blot by 48 h after transfection.

### Statistical analysis

Statistical analysis was performed using GraphPad Prism 6 (v. 6.02; La Jolla, CA). A two-way analysis of variance (ANOVA) followed by a Newmann-Keuls multiple comparisons test was used to assess differences between treatment groups of more than two factors when grouped by two variables unless otherwise indicated. Groups of two were analyzed by an unpaired t-test.

## Results

### Increased basal COX-2 protein expression in COPD-derived lung fibroblasts is not associated with a concurrent increase in Cox-2 mRNA

**Fig 1:**
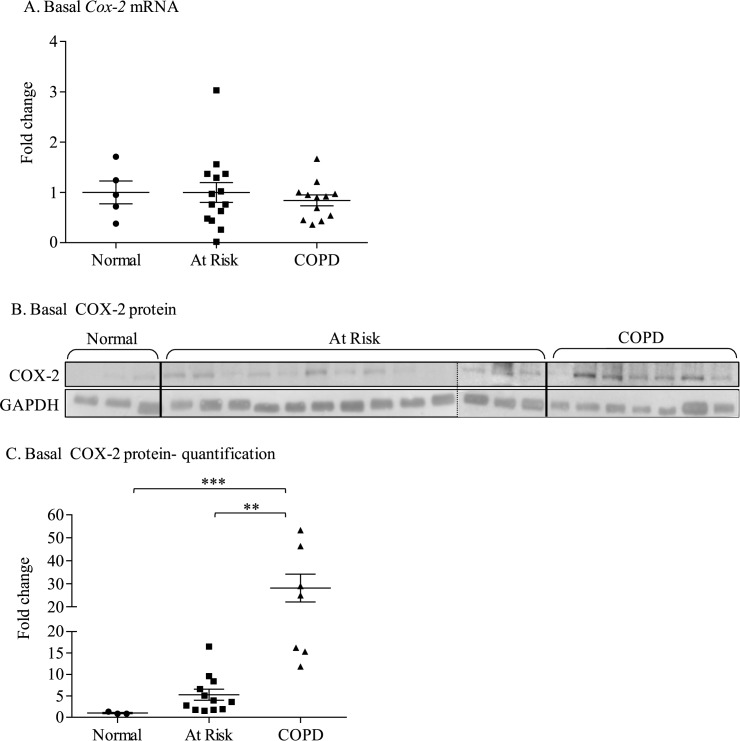
Basal *Cox-2* mRNA and protein expression in Normal, At Risk and COPD lung fibroblasts. (A) Basal *Cox-2* mRNA: There was no significant difference in basal *Cox-2* mRNA between Normal (fold change: 1 ± 0.04), At Risk (5.9 ± 2.2) and COPD fibroblasts (1.5 ± 0.69). (B) Basal COX-2 protein: Basal COX-2 levels were low in Normal (non-smoker) lung fibroblasts. A detectable increase in COX-2 protein was observed in At Risk lung fibroblasts as well as lung fibroblasts from COPD subjects. Dashed line denotes different gel. (C) Basal COX-2 protein- quantification: There was a significant increase in basal COX-2 protein expression in lung fibroblasts from COPD subjects (fold change was 28.1 ± 6.1, ** p < 0.001 compared to At Risk and *** p < 0.01 compare Normal). Results are expressed as the mean ± SEM (fold-change) of COX-2 protein levels normalized to the Normal lung fibroblasts and each symbol represents fibroblasts from a different individual.

We examined *Cox-2* mRNA and protein expression in quiescent lung fibroblasts derived from never-smokers (Normal) as well as smokers with and without COPD. *Cox-2* mRNA expression was not statistically different between the three groups (Fig 1A). Despite no difference in *Cox-2* mRNA levels, COX-2 protein expression was significantly higher in cells derived from COPD subjects (Fig 1B and 1C). These data suggest that features inherent to COPD (other than chronic smoke exposure) contribute the heightened basal COX-2 expression. These data further imply that transcriptional upregulation of the *Cox-2* gene cannot solely account for the heightened COX-2 protein expression in COPD-derived lung fibroblasts.

### Reduced AhR levels augment COX-2 protein expression in lung structural cells without alterations in mRNA levels

**Fig 2:**
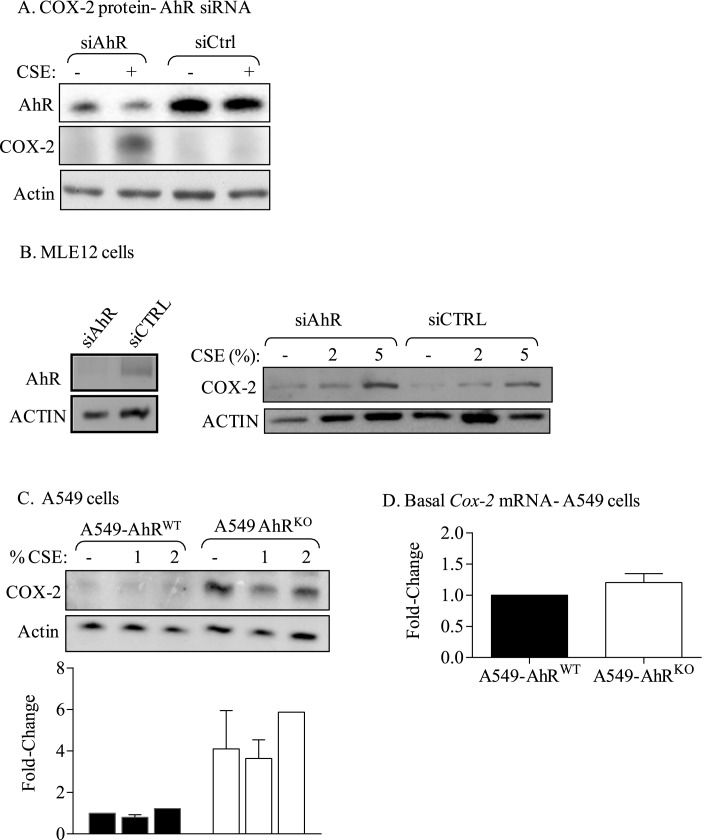
Reduction in AhR protein expression is accompanied by increased basal COX-2 protein expression in lung structural cells. (A) COX-2 protein- AhR siRNA: reduction in AhR expression in Normal human lung fibroblasts increased COX-2 protein expression. (B) COX-2 protein- MLE-12 cells: There was a significant reduction in AhR via siRNA; this was accompanied by an increase in both basal and CSE-induced COX-2 protein. (C) A549 cells: There was a robust increase in basal COX-2 protein in A549-AhR^KO^ cells. (D) *Cox-2* mRNA- A549 cells: There was no change in basal *Cox-2* mRNA levels due to deletion of the AhR in A549 cells. Results are expressed as mean ± SEM of 2–3 independent experiments.

We have shown that COPD lung fibroblasts have less AhR protein compared to Normal and At Risk fibroblasts [9], leading us to speculate that low AhR is why there is more COX-2 in COPD. To test this, we first reduced AhR expression in normal human lung fibroblasts (which expresses abundant AhR protein) [9], which resulted in an increase in COX-2 (Fig 2A). Next, we used MLE-12 cells, a distal bronchiolar and alveolar epithelial cell line, and knocked-down AhR; this also resulted in an increase COX-2 (Fig 2B). Finally, we used A549 cells, a human pulmonary adenocarcinoma cell line that exhibits features typical of type II alveolar epithelial cells [25], in which AhR was eliminated [18]. Elimination of the AhR in the A549 cells robustly increased COX-2 protein (Fig 2C), but without a concomitant change in basal *Cox-2* mRNA expression (Fig 2D). These data support that higher COX-2 protein is due to low AhR in multiple lung cell types, and that this is not due to a concomitant increase in *Cox-2* mRNA expression.

### Regulation of COX-2 in lung structural cells is due to AhR-dependent destabilization of Cox-2 mRNA

**Fig 3:**
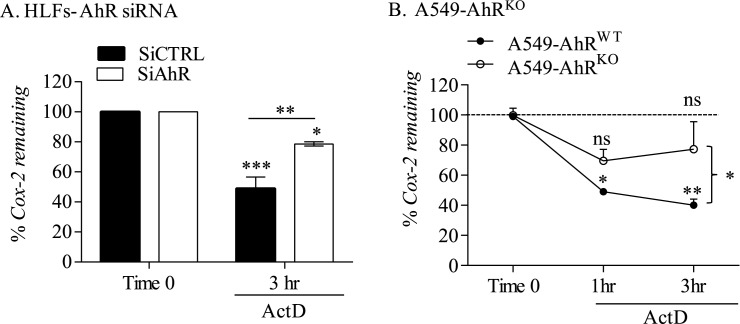
Regulation of IL-1β induction of COX-2 protein in human lung fibroblasts by the AhR is at the level of mRNA stability. (A) HLFs- AhR siRNA: There was significantly more *Cox-2* mRNA remaining after induction with IL-1β in AhR knock-down cells. (B) A549-AhR^KO^: There was a significant decline in the percentage (%) of *Cox-2* mRNA remaining within one hour after addition of ActD in A549-AhR^WT^ cells (* p < 0.05; ** p< 0.01) but not in A549-AhR^KO^ cells (ns). Results are expressed as mean ± SEM of 2 independent experiments.

We have previously shown that the AhR destabilizes *Cox-2* mRNA [4]. Given that COPD lung fibroblasts have enhanced *Cox-2* mRNA stability [11] but lower AhR [9], we speculated that enhanced *Cox-2* mRNA stability in COPD lung cells was due to low AhR expression. Following reduction in AhR levels by siRNA, Normal lung fibroblasts were exposed to IL-1β for 3 hours followed by treatment with ActD, an inhibitor of RNA synthesis [23]. In these experiments we used IL-1β, a potent inducer of COX-2 in fibroblasts [22, 26] and a cytokine that does not exhibit AhR agonist activities (data not shown) [27]. These results show that there was a significant reduction in *Cox-2* mRNA stability after treatment with ActD, the effect of which was attenuated by AhR knockdown (Fig 3A). There was similarly a rapid reduction of *Cox-2* mRNA after administration of ActD in A549-AhR^WT^ cells- but not A549-AhR^KO^ cells- exposed to IL-1β (Fig 3B). These data support that the AhR destabilizes the *Cox-2* transcript, such that low AhR in COPD-derived cells may stabilize *Cox-2* mRNA, thereby leading to increased COX-2 protein levels.

### The AhR does not control the induction of miR-146a

**Fig 4:**
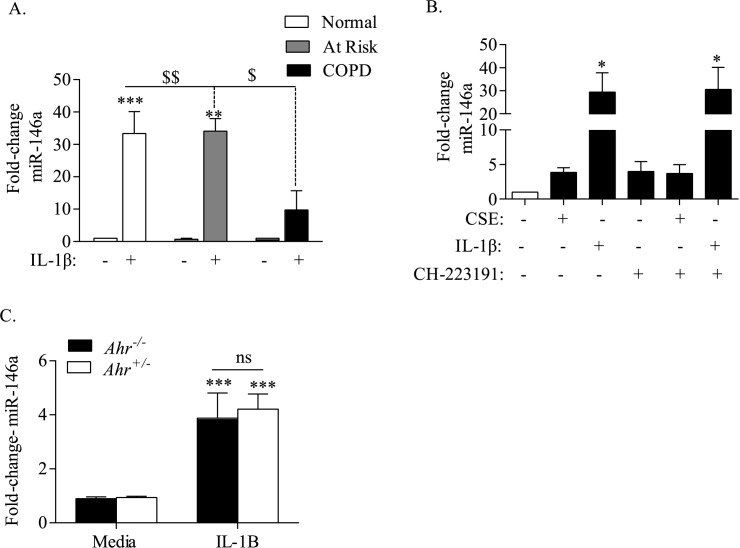
The AhR does not control miR-146a expression in response to CSE or IL-1β. (A) COPD-derived lung fibroblasts have less miR-146a in response to IL-1β- Human lung fibroblasts from Normal, At Risk or COPD subjects were exposed to IL-1β (10 ng/ml) for 6 hours and miR-146a evaluated by qRT-PCR. IL-1β significantly increased miR-146a expression in Norma (fold-increase 33.4 ± 6.8; ***p < 0.0001) and At Risk (34.1 ± 3.9; ** p < 0.01) lung fibroblasts. There was no significant induction in miR-146a in COPD fibroblasts (fold-increase 9.8 ± 5.9; $ p < 0.05, $$ p < 0.01 compared to At Risk or Normal fibroblasts, respectively). Results are expressed as the mean ± SEM, n = 3 independent experiments of samples utilizing lung fibroblasts derived from 3–6 different individuals. (B) There was a significant increase in miR-146a in human lung fibroblasts exposed to IL-1β for 6 hours (fold-increase 29 ± 8.5). There was a slight increase with CH-223191 for 6 hours but no effect when both CH-223191 and IL-1β were combined (fold change 31 ± 9.6). Results are expressed as the mean ± SEM, n = 3 separate experiments. (C) *Ahr*^*+/-*^ and *Ahr*^*-/-*^ cells were exposed to IL-1β and miR-146a levels assessed by qRT-PCR. There was a significant induction of miR-146a upon stimulation with IL-1β (*** p < 0.001); there was no significant difference in the magnitude of induction between *Ahr*^*+/-*^ and *Ahr*^*-/-*^ fibroblasts (ns). Results are expressed as the mean ± SEM, n = 12 separate experiments.

The miRNA miR-146a targets *Cox-2* mRNA for degradation and/or translation inhibition [22], making it plausible that the AhR control over miR-146a levels is why there is enhanced *Cox-2* mRNA degradation. Following exposure to IL-1β for 6 hours, there was a significant increase in miR-146a in fibroblasts derived from Normal and Smoker subjects. However, the induction of miR-146a was significantly less in COPD-derived lung fibroblasts (Fig 4A). To evaluate whether the AhR was responsible for the higher induction of miR-146a in Normal and Smoker fibroblasts (which express comparable levels of the AhR) [9], we first used a pharmacological approach to inhibit the AhR. The specific AhR antagonist CH-223191 [18, 28] had a minimal effect on miR-146a expression levels (Fig 4B). IL-1β significantly increased miR-146a, but co-exposure to CH-223191 and IL-1β did not significantly impact the ability of IL-1β to increase miR-146a expression (Fig 4B). There was also no effect of AhR inhibition with CH-223191 on the levels of miR-146a caused by CSE exposure (Fig 4B). To complement these findings, we utilized fibroblasts from *Ahr*^*+/-*^ and *Ahr*^*-/-*^ mice exposed to IL-1β. The relative level of miR-146a induction by IL-1β was 4.2 ± 0.6 and 3.9 ± 0.9, respectively, for both *Ahr*^*+/-*^ and *Ahr*^*+/-*^ cells, with there being no significant difference between the *Ahr*^*+/-*^ and *Ahr*^*+/-*^ cells (Fig 4C). Thus, we conclude that the AhR does not regulate miR-146a expression in lung fibroblasts, making it unlikely that the differences in *Cox-2* mRNA stability are due to AhR-dependent regulation of the miR-146a.

### The AhR does not control RelB induction by inflammatory stimuli

**Fig 5:**
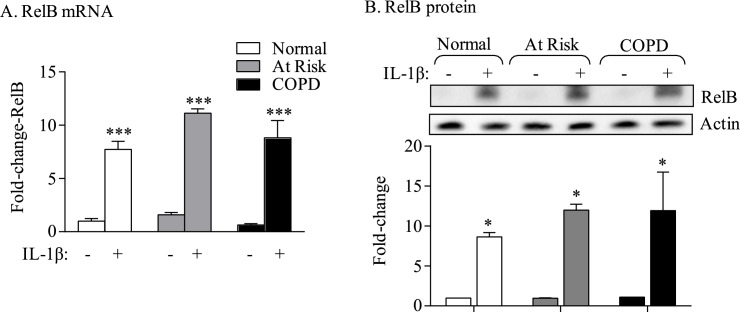
RelB expression in increased in lung fibroblasts in response to IL-1β. Human lung fibroblasts were cultured with 10 ng/ml of rhIL-1β for 6 or 24 hours and RelB mRNA assessed by qRT-PCR and cell lysate for detection of RelB protein by western blot. (A) RelB mRNA- There was a significant increase in RelB mRNA in lung fibroblasts derived from Normal, At Risk and COPD subjects compared to respective unstimulated controls (*** p < 0.001). Results are expressed as the mean ± SEM of three strains from each subject group. (B) RelB protein- There was a noticeable and significant increase in RelB protein expression upon stimulation of lung fibroblasts with IL-1β for 24 hours (*p < 0.05 for each fibroblast group compared to their respective unstimulated controls). There was no difference in RelB induction between the three groups. Results are expressed as the mean ± SEM of three independent experiments.

**Fig 6:**
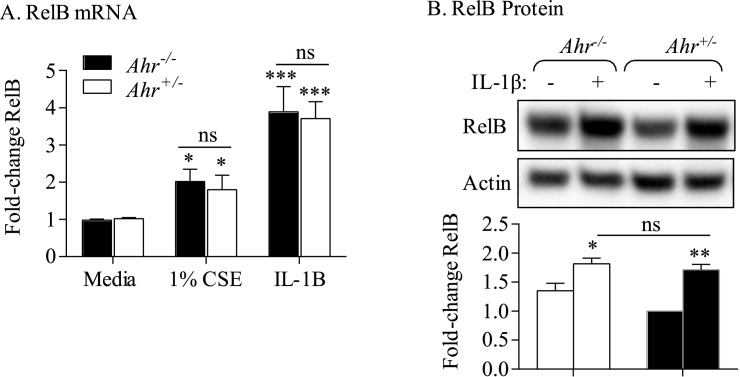
The AhR does not control RelB induction by CSE or IL-1β. *Ahr*^*+/-*^ and *Ahr*^*-/-*^ mouse lung fibroblasts were exposed to 1% CSE or rmIL-1β and RelB mRNA (A) and protein (B) evaluated by qRT-PCR or western blot respectively. There was no significant difference in RelB mRNA induction between *Ahr*^*+/-*^ and *Ahr*^*-/-*^ cells. Results are expressed as the mean ± SEM, n = 8–10 separate experiments.

The anti-inflammatory abilities of the AhR against cigarette smoke-induced COX-2 expression require RelB expression [8], leading us to speculate that the AhR-dependent control over RelB expression is how the AhR attenuates excessive COX-2 levels. For these experiments, we utilized IL-1β to induce COX-2- rather than CSE- to avoid cigarette smoke-induced proteolytic degradation of RelB [14]. Lung fibroblasts from all three subject groups exposed to IL-1β significantly increased RelB mRNA and protein expression, with there being a similar magnitude of induction between the three groups (Fig 5A and 5B), suggesting that induction of RelB was not impaired in COPD-derived lung cells. This also suggested that the AhR does not contribute to RelB induction by IL-1β. To confirm that the AhR is not involved, we utilized lung fibroblasts from *Ahr*^*+/+*^ and *Ahr*^*+/-*^ mice. Exposure to either CSE or IL-1β increased RelB mRNA levels (Fig 6A and 6B). However, there was no significant difference in the relative induction of RelB mRNA or protein in response to either CSE or IL-1β between *Ahr*^*+/+*^ and *Ahr*^*+/-*^ fibroblasts (Fig 6A and 6B). Thus, we conclude that the AhR does not appreciably control the induction of RelB expression in response to inflammatory stimuli.

### RelB expression is necessary for the transcriptional induction of Cox-2 mRNA and miR-146a in human lung fibroblasts

**Fig 7:**
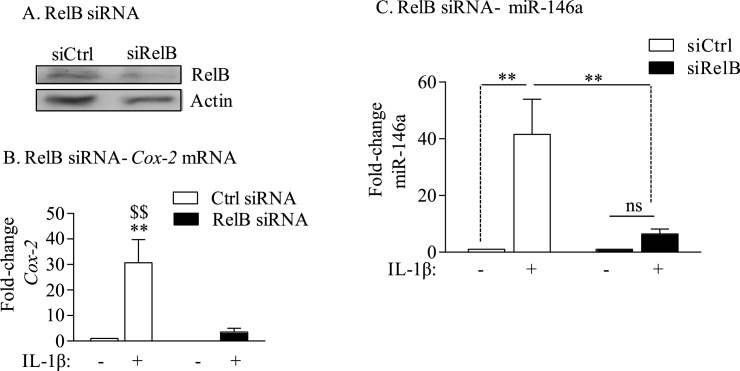
RelB regulates *Cox-2* transcription in human lung fibroblasts. (A) RelB siRNA: knockdown of RelB in Normal (non-smoker) human lung fibroblasts was approximately 50%. Representative western blot is shown. (B) RelB siRNA-*Cox-2* mRNA: There was a significant reduction in the relative level of *Cox-2* mRNA induction by IL-1β in RelB knock-down cells (** p < 0.01 compared to media-only Ctrl siRNA; $$ p < 0.01 compared to IL-1β-treated RelB siRNA). Results are expressed as the mean ± SEM of three independent experiments. (C) miR-146a- IL-1β RelB siRNA: Normal lung fibroblasts were subjected to RelB knock-down as described above and exposed to IL-1β for 6 hours. Total RNA was collected for analysis of miR-146a by qPCR. There was a significant induction in miR-146a in control lung fibroblasts exposed to IL-1β receiving the Ctrl siRNA (fold-induction: 41.6 ± 12.3; *p < 0.05). Knock-down of RelB significantly attenuated miR-146a induction (fold induction: 6.4 ± 1.8; $p < 0.05 compared to Ctrl-siRNA). Results are expressed as the mean ± SEM, n = 5 separate experiments.

Our data show that the AhR does not control the transcription of *Cox-2* in response to CSE or IL-1β (Figs 2 and 3). Our previous data in mouse lung fibroblasts suggest that RelB is necessary for optimum induction of *Cox-2* mRNA [22]. Knock-down of RelB in Normal human lung fibroblasts using siRNA (Fig 7A) significantly impaired the induction of *Cox-2* mRNA expression when cells were exposed to IL-1β (Fig 7B, *black bars*). In cells which received control siRNA (*i*.*e*. with RelB expression; siCtrl), there remained robust induction of *Cox-2* mRNA (Fig 7B, *open bars*). These data support that RelB controls the transcriptional regulation of *Cox-2* mRNA in human lung fibroblasts. We also evaluated whether the ability of IL-1β to induce miR-146a depended on RelB expression. Our data show that knock-down of RelB significantly attenuates the ability of IL-1β to increase miR-146a (Fig 7C). Thus, these data show that RelB controls miR-146a expression in response to inflammatory cytokines, and when considered as a whole, supports that both the AhR and RelB contribute to the suppression of COX-2 by two independent but complementary pathways.

## Discussion

COPD is an obstructive lung disease that is increasing in prevalence worldwide, affecting an estimated 200 million people [29]. While the etiology of COPD is strongly linked to smoke exposure, the underlying pathogenic mechanisms by which smoke causes chronic, aberrant pulmonary inflammation remains poorly defined. The purpose of this study was to further understand how the AhR suppresses COX-2 expression in association with COPD. We have published that two signalling pathways involving the NF-κB protein RelB and the AhR diminish the expression of inflammatory mediators, including COX-2, caused by cigarette smoke exposure [8, 30]. We had postulated that AhR-dependent induction of miR-146a serves as a post-transcriptional regulatory mechanism for the attenuation of COX-2 protein expression. A significant component of this was based on our intriguing observations that lung fibroblasts from COPD subjects expressed significantly more basal COX-2 protein compared to fibroblasts derived from either At Risk or Normal subjects (Fig 1). This result is consistent with a report by Togo and colleagues who demonstrated heightened COX-2 protein expression in COPD lung fibroblasts compared to fibroblasts derived from smokers [31]. We extended this finding by providing further evidence that this increase in COX-2 is an inherent feature not due to smoke exposure alone, as there was no difference in COX-2 between cells from Normal subjects compared to At Risk subjects. These data also highlight that the heightened COX-2 protein in COPD-derived lung fibroblasts was not the result of heightened *Cox-2* mRNA expression (Fig 1), implying that basal COX-2 protein levels in COPD lung cells are controlled by mechanisms independent from direct transcriptional regulation. To understand the basis of this, we turned our attention to the AhR, as we have recently shown there is less AhR protein in COPD lung fibroblasts [9]. Using complementary techniques in lung structural cells, we confirmed that loss of AhR expression contributes to increased COX-2 protein without a concomitant increase in *Cox-2* mRNA levels, supporting the notion that homeostatic control over COX-2 protein- in the absence of exogenous inflammatory stimuli- is dependent on AhR expression.

Our data also suggest that the extent to which the AhR controls basal versus induced (e.g. CSE or IL-1β) COX-2 may be at least partially related to the absolute level of AhR. We have previously shown that AhR protein levels are reduced by approximately 80% in COPD-derived lung fibroblasts, a decrease that is sufficient enough to eliminate induction of the target gene *Cyp1b1* [9]. Likewise, our data show that elimination of AhR levels by zinc finger nuclease technology (A549-AhR^KO^) prevents *Cyp1a1* expression in A549 cells [18]. In both the COPD-derived lung fibroblasts and A549-AhR^KO^ cells, this decrease in AhR expression was sufficient enough to increase basal COX-2 protein (Figs 1 and 2). The fact that there was no increase in COX-2 in the A549 cells exposed to CSE may reflect their inherent insensitivity to cigarette smoke [32]. In the Normal human lung fibroblasts, there was no increase in basal COX-2 after knockdown with siAhR (Fig 1A), a finding we speculate may be due to sufficient remaining AhR expression. The increase in COX-2 in the mouse lung epithelial cells (MLE-12 cells)- irrespective of AhR expression- differs from the primary lung fibroblasts (Fig 2A and 2B). This may be due to inherent differences in cell type (epithelial versus fibroblast) or species (mouse versus human). Despite some slight differences in expression patterns, our data highlight that the AhR suppresses COX-2 levels in lung structural cells.

Typically the induction of COX-2 is transient and returns to baseline levels within 24–28 hours. Post-transcriptional regulation of protein expression is an adaptive mechanism that is crucial in regulating the timing and the amount of inflammatory proteins including COX-2. Although the *Cox-2* gene is transcriptionally-controlled (*e*.*g*. via NF-κB in response to IL-1β [33] or CSE [26]), and mechanisms such as protein turnover contribute to COX-2 expression [34], the level of COX-2 protein is determined in large part by changes in the half-life of the mRNA Thus, there is often a poor correlation between *Cox-2* mRNA and protein levels because *Cox-2* mRNA is rapidly degraded [4], raising the possibility that mRNA degradation could be why there is discord between mRNA and protein levels in COPD lung fibroblasts. Not only is COX-2 a target of miR-146a but there is significantly impaired induction of miR-146a in COPD-derived cells in response to inflammatory stimuli (Fig 4). The AhR contributes significantly to the regulation of miRNA [18, 35–37], which led us to postulate that a key component to the regulation of COX-2 by the AhR would be induction of miR-146a. However, as we were unable to detect differences in miR-146a levels using AhR-deficient cells or with the AhR antagonist CH-223191, we ultimately concluded that the AhR does not appreciably control miR-146a expression.

We do show that down-regulation of RelB via siRNA decreases the magnitude of IL-1β-inducd miR-146a expression (Fig 7) despite the fact the RelB was significantly increased in all three subject groups. We therefore conclude that RelB-but not the AhR- contributes to the induction of miR-146a. RelB is a part of the non-canonical NF-κB pathway that is involved in thymic and secondary lymphoid organogenesis as well as B cell development [38]. RelB expression is increased by inflammatory stimuli, which may serve as negative feedback loop to attenuate the transcriptional abilities of the classic NF-κB pathway [39]. Importantly, RelB prevents persistent non-infectious inflammation in the liver and lung, phenomena attributed to the suppressive abilities of RelB in non-lymphoid tissue, possibly fibroblasts [40, 41]. Even though there is transcriptional dependence by RelB on the induction of *Cox-2* mRNA, our previous data show that RelB maintains profound control over the amount of COX-2 protein that is ultimately expressed [22]. In this regard, RelB may have a dual role by both dictating *Cox-2* mRNA induction in addition to transcribing for miR-146a, a feature that would post-transcriptionally keep COX-2 protein levels in check.

We propose that both AhR and RelB are necessary to control an aberrant inflammatory response. The true mechanism by which the AhR controls COX-2 protein remains unclear but could involve other post-transcriptional mechanisms involving human antigen R (HuR), an RNA binding protein recently shown by us to be regulated by the AhR as a means to down-regulate COX-2 levels [4]. It is possible that the RelB-dependent induction of miR-146a impairs the ability of HuR to stabilize target mRNA, including COX-2. This notion is support by evidence showing that HuR is a direct target of miR-146 [42]. Whether HuR is decreased by RelB-dependent induction of miR-146a or whether HuR is involved in the AhR-dependent regulation of COX-2 mRNA versus protein in COPD lung cells are currently active areas of investigation.

In conclusion, our data support that the AhR suppresses COX-2 expression and that both AhR and RelB may work cooperatively to suppress COX-2 expression in response to inflammatory stimuli. This is not an unreasonable assumption given that in a model of immune tolerance, miR-146a enhances RelB binding to the promoter of inflammatory genes, resulting in transcriptional silencing [43]. Our data further support the notion that dysregulated expression levels AhR and RelB play an important mechanistic role in the development and progression of smoke-induced pathologies such as COPD. We cannot yet conclude that low AhR and RelB levels in COPD [9, 14] predisposes to the development of COPD or foresees rapid declines in lung function. Further, we do not anticipate that RelB could be used as a general blood-based biomarker of COPD, as systemic RelB expression is similar between Normal, Smokers and COPD subjects [14]. However, RelB levels changed with exacerbations in COPD subjects and predicted changes in arterial stiffness, a measure of cardiovascular risk [44]. While our investigations into systemic AhR levels in COPD are currently underway, it is interesting to speculate that, together with FEV_1_/FVC, AhR and RelB may be a useful diagnostic tool for COPD and associated co-morbidities. Finally, understanding if AhR and RelB levels in the lung are inherently low (*i*.*e*. not due to disease progression) could also form the basis for anti-inflammatory therapies targeting an AhR-RelB axis in COPD, a disease with few options to treat the underlying inflammation that may drive disease progression.
